# Supplementary material for: Association between oral microbiome and seven types of cancers in East Asian population: a two-sample Mendelian randomization analysis
Source: Front Mol Biosci. 2023 Nov 21;10:1327893. doi: 10.3389/fmolb.2023.1327893 (PMC10702768; doi:10.3389/fmolb.2023.1327893)
Supplement: Supplementary file 1 [file DataSheet1.ZIP › Supplementary Materials/MR plots for tongue/tongue═╝/Ovarian cancer/pheno.1206_to_ovarian cancer_forest.pdf]

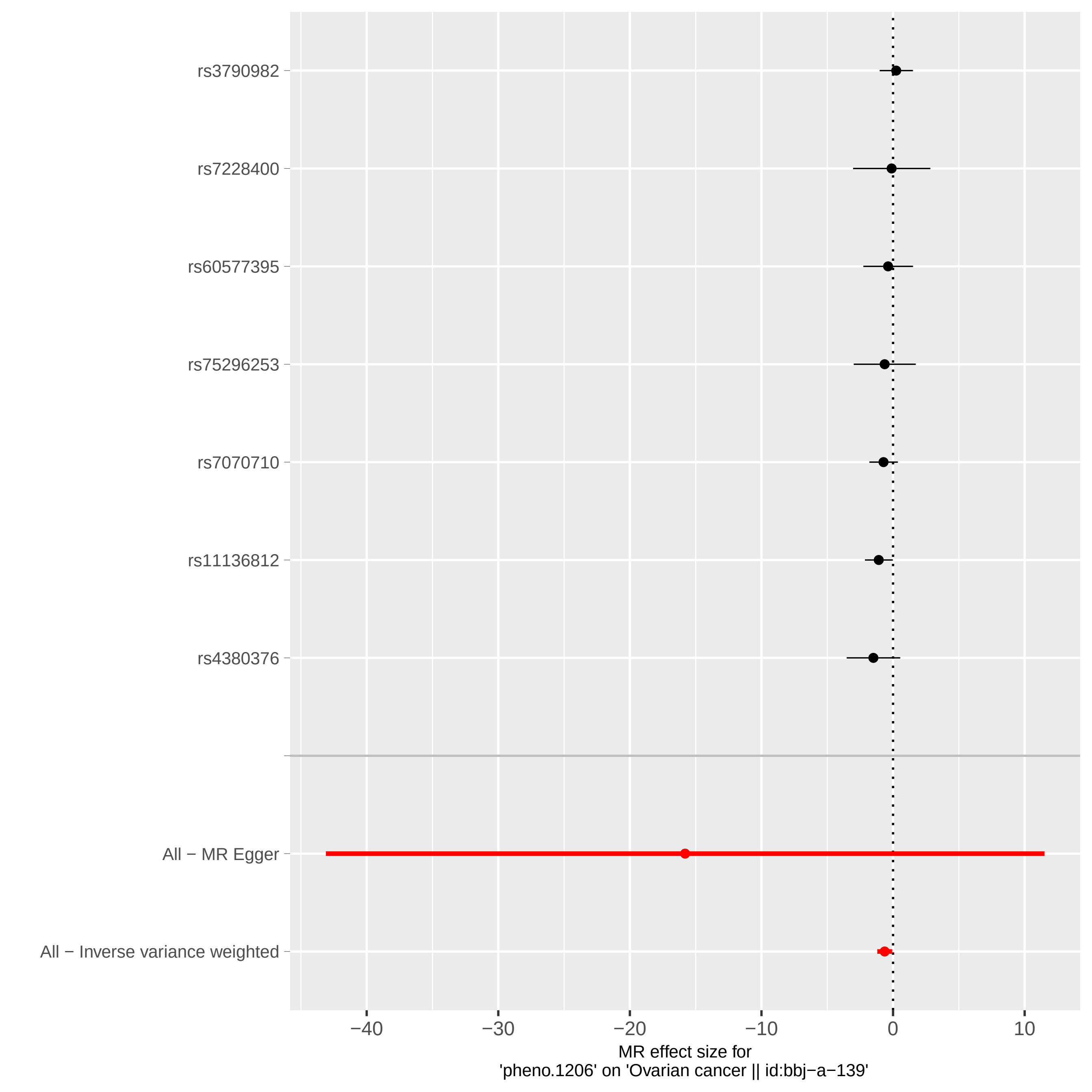
rs3790982
rs7228400
rs60577395
rs75296253
rs7070710
rs11136812
rs4380376
All − MR Egger
All − Inverse variance weighted
−40
−30
−20
−10
0
10
MR effect size for
'pheno.1206' on 'Ovarian cancer || id:bbj−a−139'
